# Development and Internal Evaluation of an Integrated Patient–Embryo–Endometrial Scoring System for Predicting Biochemical Pregnancy in IVF: A Proof-of-Concept Pilot Study

**DOI:** 10.3390/diagnostics16162508

**Published:** 2026-08-09

**Authors:** Egehan Bilen, Sultan Seda Doğan, Müge Kovalı Sezer, Cansu Tuğçe Çetinbaş Açar, Emre Okyay

**Affiliations:** 1 Division of Reproductive Endocrinology and Infertility (IVF Center), Department of Obstetrics and Gynecology, Dokuz Eylül University School of Medicine, Balçova, 35340 Izmir, Turkey; 2 Department of Obstetrics and Gynecology, İzmir Buca Seyfi Demirsoy Training and Research Hospital, 35390 Izmir, Turkey; 3 Division of Perinatology, Department of Obstetrics and Gynecology, Ege University Faculty of Medicine, 35100 Izmir, Turkey

**Keywords:** in vitro fertilization, endometrial receptivity, embryo quality, implantation prediction, scoring system, ultrasonography

## Abstract

**Background/Objectives:** Implantation in in vitro fertilization (IVF) depends jointly on patient prognosis, embryo quality and endometrial receptivity, yet these are usually assessed in isolation. We developed and internally evaluated, as a proof of concept, an index combining all three. **Methods:** In this single-center prospective pilot study, 46 women undergoing embryo transfer were scored on the day of transfer for patient prognosis (SART model), embryo morphology and endometrial receptivity (Endo-R ultrasound score), each on a 0–10 scale and combined as (SART/10) × (Embryo + Endo-R)/2. The outcome was biochemical pregnancy (10 events). Discrimination (area under the curve, AUC), internal validation and incremental value beyond age were assessed by bootstrap resampling, cross-validation and likelihood-ratio tests. **Results:** The Integrated Score achieved the highest discrimination of the indices examined (AUC 0.86, 95% CI 0.71–0.98; cross-validated 0.80) but did not significantly outperform age alone (0.79; *p* = 0.33), and its embryo component contributed none (0.51; χ^2^ = 0.42, *p* = 0.52 when added to age). Only the difference from the Embryo Score survived Bonferroni correction. The Endo-R Score added information beyond both age and the SART Score (both *p* = 0.003). **Conclusions:** The index did not add measurable discrimination beyond patient age in this sample; the endometrial component carried independent information and is the domain worth pursuing. The prognosis model was applied outside its validated endpoint and population, so it ranks rather than calibrates risk. With 10 events these estimates are exploratory and the Youden-derived cut-off (≥4.2) is optimistic. The study contributes a specified rubric and open calculator for the multicenter validation that must follow.

## 1. Introduction

Infertility affects approximately one in six couples of reproductive age, representing a significant public health concern [1]. Against a background of declining global fertility rates [2] and a rising demand for assisted reproduction, improving the efficiency of in vitro fertilization (IVF) has substantial individual and societal importance. Despite substantial advances in personalized ovarian stimulation, extended embryo culture, preimplantation genetic testing, and embryo selection, success rates remain modest: in the most recent European registry data, clinical pregnancy was achieved in 22.1% of IVF and 20.0% of ICSI cycles per oocyte retrieval, and in approximately one-third of embryo transfers [3]. Successful implantation requires a receptive endometrium, good-quality embryos, and synchronized embryo–endometrial dialog [4]. Female age remains the dominant determinant of success in contemporary practice: in a five-year cohort of more than 5,000 couples undergoing a first IVF cycle at public centers in Portugal, patient demographic characteristics, female age foremost, drove most of the variation in outcome [5].

Age, however, is fixed by the time treatment begins. Among the determinants that can still be assessed on the day of transfer, endometrial receptivity is the least well characterized. Endometrial receptivity, the transient state in which the endometrium becomes able to accept an implanting embryo through precise synchronization with embryonic development, remains one of the most challenging aspects of IVF to assess accurately [6]. The human endometrium is receptive to the embryo during a specific period known as the window of implantation (WOI), typically occurring between days 19 and 21 of a 28-day menstrual cycle [6,7]. Embryo factors are estimated to account for about one-third of implantation failures, with suboptimal endometrial receptivity and altered embryo–endometrial dialog together being responsible for the remaining two-thirds [8].

Conventional assessment of endometrial receptivity relies on manual measurement of various parameters observed through ultrasound examination, including endometrial pattern, thickness, uterine cavity volume, and uterine blood flow [8]. However, interpretation of these parameters is fraught with variability and subjectivity. Notably, the measurement of endometrial thickness, a key parameter in assessing receptivity, lacks a universally accepted gold standard, leading to inconsistencies in clinical practice [8,9]. Individual ultrasound markers of endometrial receptivity characteristically demonstrate high sensitivity but low specificity [8], suggesting that alternative assessment approaches may be needed.

Several scoring systems have been proposed to standardize the assessment of uterine receptivity. The Applebaum uterine scoring system assigns points across seven sonographic parameters, comprising endometrial thickness, endometrial layering, myometrial contractions, myometrial echogenicity, uterine artery pulsatility index, zone 3 endometrial blood flow, and myometrial blood flow, to give a composite 0–20 assessment of uterine receptivity [10]. Its cut-off values were presented as preliminary by the author, who anticipated that they would evolve as further patients were assessed. A modified version of this scoring system has been applied specifically in frozen embryo transfer cycles with promising results [11]. However, these systems focus exclusively on uterine parameters and do not incorporate embryo quality or patient-specific prognostic factors.

Meanwhile, embryo morphological assessment, using the Baczkowski scoring system for cleavage-stage embryos and the Gardner grading system for blastocysts, provides an independent evaluation of embryo implantation potential [12,13]. Patient-specific predictive models built on the United States Society for Assisted Reproductive Technology (SART) registry incorporate demographic and clinical factors to estimate the cumulative probability of a live birth across IVF cycles [14].

Critically, these three domains are not independent. Even a top-quality embryo cannot implant in a non-receptive endometrium, and a receptive endometrium cannot compensate for a poor embryo or an unfavorable patient prognosis; implantation behaves as a conjunctive process in which every domain must be simultaneously favorable. A single excellent parameter, such as a high embryo grade, is therefore reassuring only when the endometrium and the patient’s prognosis are also favorable. This reasoning argues against the common practice of judging the endometrium or the embryo in isolation, and in favor of an integrated assessment in which any one unfavorable domain pulls down the overall predicted likelihood of success.

Although clinicians routinely weigh patient prognosis, embryo quality, and endometrial appearance together when counseling patients, and predictive models combining subsets of these factors exist [9], to our knowledge these three domains have not previously been combined into a single explicit, quantitative index for implantation outcomes. The purpose of this pilot study was to develop and internally evaluate such an integrated scoring system and to compare its predictive performance against its individual components and established scoring methods. A second, deliberate objective was to make the instrument reusable irrespective of that outcome. Every scoring rule is specified in full, and the index is implemented as an openly available calculator with published source code, so that the multicenter derivation and validation study needed to settle the question can be executed against a common scoring standard.

## 2. Materials and Methods

### 2.1. Study Design and Population

This single-center prospective pilot study was conducted at the Reproductive Endocrinology and Infertility Unit, Department of Obstetrics and Gynecology, Dokuz Eylül University Hospital, Izmir, Turkey, between March and December 2023. The study was approved by the Dokuz Eylül University Non-Interventional Research Ethics Committee (protocol number: 7930-GOA; decision number: 2023/09-17; approval date: 22 March 2023), under which the study was conducted; a subsequent title-change amendment was approved on 10 January 2024 (decision number: 2024/02-20). Written informed consent was obtained from all participants. No formal sample-size calculation was performed; the study analyzed a convenience sample of all eligible cycles within the recruitment window.

All transvaginal ultrasound assessments were performed on the day of embryo transfer by a single operator (E.B.), who was not involved in the patients’ ovarian stimulation, endometrial preparation, embryo grading, or the embryo-transfer decision. Embryo morphological grades were assigned independently by the embryology laboratory, and biochemical pregnancy outcomes were obtained afterward from clinical records. Because the scoring components were derived from data collected outside the clinical decision pathway, they could not have influenced treatment or outcomes, avoiding a treatment-paradox bias. As all assessments were made by one operator, inter-observer reproducibility could not be evaluated.

#### 2.1.1. Inclusion Criteria

- Women aged ≥ 18 years undergoing an IVF cycle with embryo transfer.
- Endometrial ultrasound image obtained prior to embryo transfer.
- Endometrial image captured using standard transvaginal ultrasound imaging systems (2D or 3D).
- Image showing the entire uterus in the focal plane with the complete endometrial stripe visible from the fundal region to the endocervix in the midsagittal plane.
- Availability of stage-appropriate morphological grading sufficient to compute the Embryo Score (individual Baczkowski parameters for cleavage-stage embryos, or Gardner expansion, inner cell mass and trophectoderm grades for blastocysts).

#### 2.1.2. Exclusion Criteria

- Canceled embryo transfer cycles.
- Presence of uterine fibroids or adenomyosis.
- Any condition causing uterine shape distortion or prior surgery to correct such conditions.

### 2.2. Data Collection

The following data were collected for each participant:

**Baseline characteristics:** Age, height, weight, menstrual cycle length, parity, history of term delivery, fertility-affecting medical conditions (polycystic ovary syndrome, male factor, endometriosis, low ovarian reserve, unexplained infertility), number of previous embryo transfers, and total oocyte count.

**IVF procedure data:** Cycle type (natural or hormonal), fresh or frozen embryo transfer, embryo developmental stage at transfer (day 3 cleavage or day 5–6 blastocyst), and number of embryos transferred.

**Endometrial assessment:** Transvaginal ultrasound evaluation was performed on the morning of embryo transfer using a Voluson E8 ultrasound system with a transvaginal transducer (GE HealthCare, Chicago, IL, USA). Parameters assessed included endometrial thickness (mm), endometrial pattern, endometrial volume (mL), zone 3 endometrial blood flow, myometrial echogenicity, myometrial blood flow, uterine artery end-diastolic flow, and bilateral uterine artery pulsatility index (PI). Endometrial–subendometrial vascularity was graded by zone as scored in the Applebaum profile [10], zone 3 denoting vascular signals that penetrate the inner hypoechoic endometrial layer adjacent to the uterine cavity. Zone 3 flow was categorized as absent, present/sparse, or abundant/multifocal on power Doppler, with the point values of that profile. Endometrial volume was obtained from three-dimensional acquisition. Color and power Doppler gain and pulse-repetition-frequency settings were held constant across examinations.

**Embryo morphological assessment:** Light microscopy images of embryos were recorded on the day of transfer. For frozen embryos, images were obtained after thawing.

**Figure 1:**
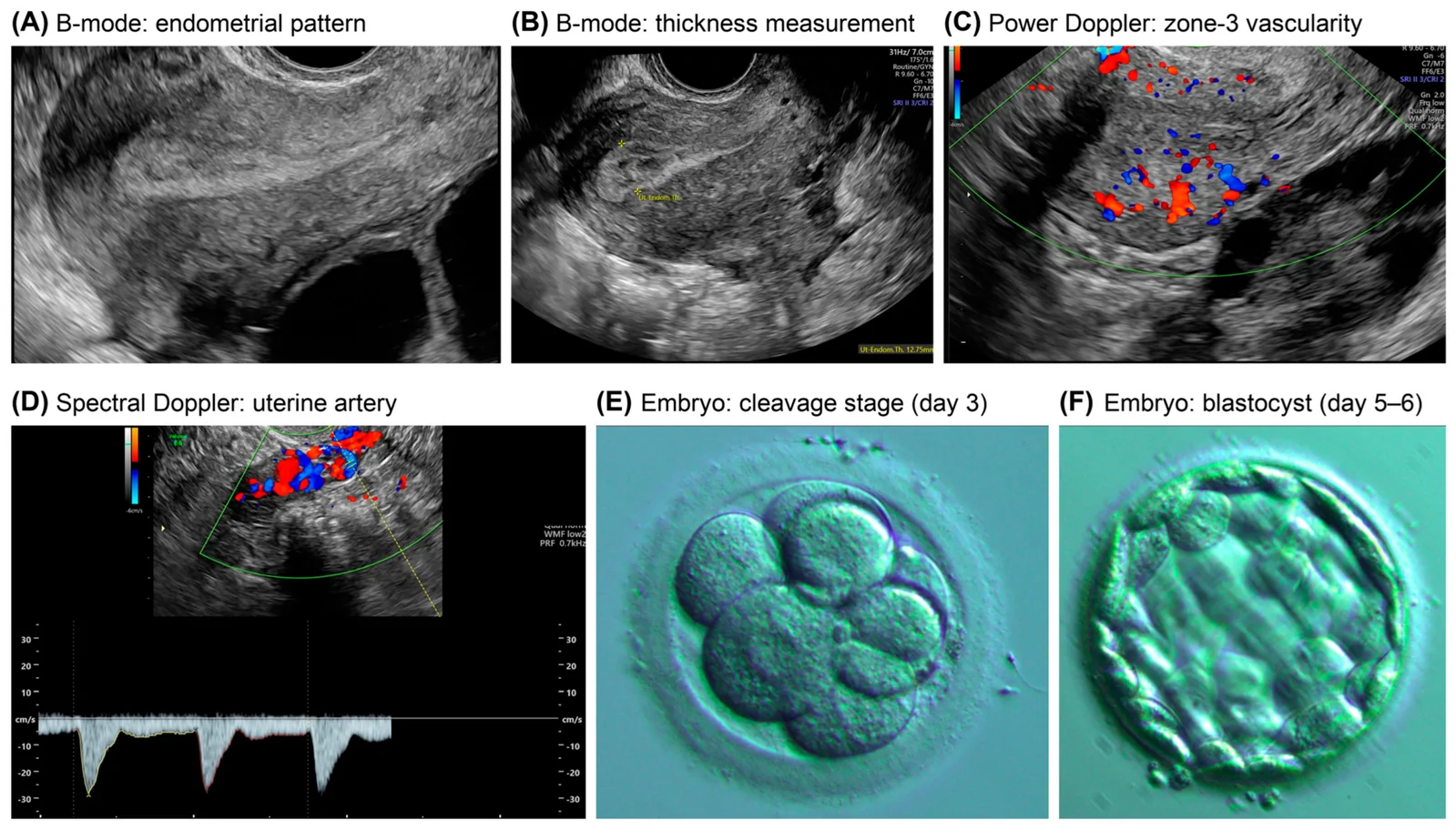
Representative de-identified imaging inputs of the Integrated Implantation Score, spanning the ultrasound and embryological parameters that feed its component scores. (**A**) B-mode transvaginal ultrasound of the endometrium in the midsagittal plane, showing the endometrial pattern. (**B**) B-mode measurement of endometrial thickness with electronic calipers. (**C**) Power Doppler image showing endometrial and subendometrial (zone 3) vascularity, graded by the Applebaum zonal classification. (**D**) Spectral Doppler of the uterine artery, from which the pulsatility index was derived. (**E**) Light-microscopy photomicrograph of a cleavage-stage embryo and (**F**) an expanded blastocyst, representing the morphology scored by the rule-based Embryo Score. Panels (**A**–**D**) were obtained on the day of embryo transfer and feed the Modified Applebaum and Endo-R Scores. All images are from the study cohort and contain no patient identifiers. Abbreviations: Endo-R, endometrial receptivity; PI, pulsatility index.

Representative de-identified study images illustrating the ultrasound and embryological basis of the score are shown in Figure 1.

### 2.3. Scoring Systems

#### 2.3.1. SART Pregnancy Probability Score (0–10)

A patient prognosis score for each woman was derived from the validated IVF prediction model of McLernon et al. [14], which was developed on the United States Society for Assisted Reproductive Technology (SART) Clinic Outcome Reporting System (CORS). The model is implemented as a freely available online calculator (available at https://w3.abdn.ac.uk/clsm/SARTIVF/, accessed on 20 June 2026). The post-retrieval version used here incorporates patient age, height, weight, infertility diagnosis, previous IVF history, and the number of oocytes retrieved. The last of these is strongly prognostic of cumulative live birth [15] and therefore carries substantial weight in the model. Its predicted percentage was linearly normalized to a 0–10 scale and is referred to throughout as the SART Score. The model’s native endpoint is cumulative live birth, so applying it to single-transfer biochemical pregnancy in a Turkish cohort places it outside both its validated endpoint and its derivation population; its output is used strictly as a relative prognostic ranking rather than as a calibrated probability.

#### 2.3.2. Embryo Morphological Quality Score (0–10)

A structured, criteria-based embryo morphological quality scoring system was developed to provide a standardized numerical assessment of embryo implantation potential. The scoring criteria differed based on embryo developmental stage:

**Cleavage-stage embryos (Day 3)** were scored based on five morphological parameters according to established Baczkowski criteria [12] (Table 1):

**Blastocyst-stage embryos (Day 5–6)** were scored based on the Gardner grading system [13,16] (Table 2):

The 0–10 scale was constructed as follows. Within each established grading system, every morphological parameter was assigned ordinal points increasing with grade quality, with relative weights pre-specified to reflect the recognized association of each parameter with implantation potential; the weight assigned to each parameter and its basis are set out in Supplementary Methods S1. Points for each stage were scaled to sum to a maximum of 10, so that cleavage and blastocyst assessments share a common 0–10 scale. The weights were fixed in advance and were not optimized against the pregnancy outcome. Morphological grades assigned by the embryologist on the day of transfer were converted to the numerical score by applying this fixed-point system deterministically.

Single-embryo transfer was performed in 20 cycles (43.5%) and double-embryo transfer in 26 (56.5%); for double transfers, the score of the higher-quality embryo was used. In four cycles (8.7%) with incompletely documented morphological sub-grades, the embryo score was imputed with the stage-specific cohort median, computed blind to pregnancy outcome. Grading system assignment followed developmental stage strictly: Baczkowski criteria [12] for cleavage-stage (day 3) embryos and Gardner criteria [13,16] for blastocysts (day 5–6). In 7 of 46 cycles (15.2%) the laboratory record for a day-5 embryo contained only a summary morphological grade rather than the separate expansion, inner cell mass and trophectoderm grades required by the Gardner system. For these cycles the cleavage-stage parameter set was applied to the available descriptors, and a sensitivity analysis restricted to cycles with complete, stage-appropriate grading is reported in Section 3.4. Mapping the two grading systems onto a common 0–10 scale assumes that a given numerical value carries equivalent implantation potential at both developmental stages, an equivalence that has not been independently established.

#### 2.3.3. Modified Applebaum Score (0–20)

The Modified Applebaum uterine scoring system was applied as previously described [10,11]; its seven parameters and their point values are given in Table 3. Six of the seven parameters, with their point values, are those of the original Applebaum profile [10]; uterine artery end-diastolic flow replaces one myometrial parameter of that profile at the same weighting, so the maximum attainable score remains 20.

#### 2.3.4. Endometrial Receptivity Score (Endo-R Score, 0–10)

A novel simplified endometrial receptivity scoring system was developed specifically for this study, incorporating five ultrasound-derived parameters (Table 4):

The five parameters were drawn from the systematic review and meta-analysis of Craciunas et al. [8], in which endometrial thickness, endometrial pattern, Doppler indices and endometrial wave-like activity were each associated with clinical pregnancy, although that review found their individual predictive accuracy insufficient for clinical use. Cut-off values were taken from the primary studies of each marker rather than from the review; the bands as implemented, a 7–14 mm thickness window and a volume of ≥2 mL, are given in Table 4. Each marker has been studied individually in relation to implantation or pregnancy outcome: endometrial thickness and pattern [8,17], endometrial volume on three-dimensional ultrasound [18], endometrial and subendometrial blood flow [19], and uterine artery impedance [20]. The evidence for uterine artery impedance is the weakest of the five, since mid-luteal pulsatility and resistance indices did not differ between women who conceived and those who did not in a prospective cohort undergoing assisted reproduction [20], which anticipates the absence of any contribution from this component here (Section 4.3). Taking thresholds from the literature rather than optimizing them within the present cohort trades some in-sample discrimination for protection against overfitting in a 46-patient sample, and it is why it is a component that performs poorly here, the uterine artery pulsatility index, was retained at its published threshold. For thickness specifically, a recent systematic review and meta-analysis in fresh and frozen–thawed transfer supports the underlying association, but describes a gradient of effect rather than a critical threshold, reporting live-birth rates above the reference category even at 14–16 mm [21]. The dichotomous band used here is therefore a simplification, and its upper bound is not established by that evidence. The literature-derived thresholds may in any case be poorly calibrated to an individual population, and cohort-specific re-derivation is an objective of the planned validation study.

#### 2.3.5. Integrated Implantation Score (0–10)

The Integrated Implantation Score was calculated by combining all three independent scoring components.

Integrated Score = (SART Score/10) × (Embryo Score + Endo-R Score)/2

The formula reflects three elements of clinical rationale: patient prognosis scales the local assessment; embryo quality and endometrial receptivity are complementary determinants of implantation and are weighted equally; and neither optimal embryo–endometrium conditions nor a favorable patient prognosis can fully compensate for the other, which is why the relationship is multiplicative rather than additive. In equation the SART Score, already on a 0–10 scale (Section 2.3.1), is divided by 10 a second time to yield a dimensionless multiplier between 0 and 1, so that the product retains the 0–10 range of its components. This structure was specified a priori on clinical grounds, before any association with pregnancy outcome was examined, and only this formulation was carried forward as the primary index; no competing formulas were tested during development. Three qualifications apply. The equal weighting of the embryo and endometrial terms is a pre-specified clinical assumption rather than a data-derived optimum, since no evidence known to us establishes a defensible weighting ratio between them. The formula is a hypothesis about how the three domains combine rather than a validated structure; a post hoc comparison against alternative structures, reported in the Supplementary Materials, shows that the present data cannot distinguish between candidate formulations. Finally, the index is an ordinal ranking rather than a probability or a proportion of an attainable maximum. Because the patient-prognosis multiplier is typically well below 1, observed values occupy only the lower part of the nominal 0–10 range, and a score of 4 must not be read as 40% of an ideal result or as a 40% chance of pregnancy. Deriving empirical weights and testing formula structure require a far larger dataset and constitute the first objective of the planned derivation study.

All three component scores and the integration formula were implemented in a freely available, self-contained, browser-based calculator whose source code is openly released on GitHub (Prototype v0.3: Figure S1) [22]. The calculator computes each component score from its input parameters and combines them into the Integrated Score, allowing the rubric to be inspected interactively.

### 2.4. Outcome Measures

The primary outcome was biochemical pregnancy, defined as a positive serum β-hCG test approximately 12 days following embryo transfer. All predictive performance analyses reported in this study refer to this biochemical pregnancy endpoint.

### 2.5. Statistical Analysis

Continuous variables were expressed as mean ± standard deviation (SD) and compared between groups using the independent samples *t*-test, with Welch’s correction applied where the assumption of equal variances was not met. Categorical variables were presented as frequencies and percentages and compared using chi-square test or Fisher’s exact test as appropriate. Correlation between scoring systems was assessed using Pearson’s correlation coefficient.

The predictive performance of each scoring system was evaluated using receiver operating characteristic (ROC) curve analysis, with area under the curve (AUC) as the primary metric. Because the scoring system was both developed and tested within a single cohort, the apparent (in-sample) performance is optimistically biased; internal validation of the Integrated Score was therefore performed by leave-one-out and repeated (200×) stratified 5-fold cross-validation, and the cross-validated AUC is reported as the more realistic estimate. Model development and reporting followed the applicable items of the TRIPOD recommendations for multivariable prediction models [23] and their updated guidance [24]; a completed checklist is provided as Supplementary Table S1. Given the small sample and low event count, evaluation focused on discrimination and internal validation, and a formal calibration analysis was not undertaken. Ninety-five percent confidence intervals (CIs) for the AUC were estimated by bootstrap resampling (2000 replicates), and the optimal cut-off of the Integrated Score was identified using the Youden index, at which sensitivity, specificity, and predictive values were calculated. Differences between paired AUCs, including the Integrated Score versus each individual component and versus patient age alone, were tested by bootstrap resampling (4000 replicates); because five such comparisons were performed, Bonferroni-corrected *p*-values are reported alongside uncorrected values. The incremental contribution of each component beyond patient age was assessed by likelihood ratio tests comparing nested logistic models. Logistic regression was also performed as a check of the joint contribution of the components. Statistical significance was set at *p* < 0.05. The likelihood-ratio tests were not pre-specified, and the multivariable models are limited by the event count, so both are reported as exploratory; *p*-values elsewhere are presented without adjustment for multiple comparisons unless otherwise stated. Descriptive statistics and group comparisons were performed in SPSS version 24.0 (IBM Corp., Armonk, NY, USA); cross-validation, bootstrap confidence intervals, and AUC comparisons were performed in Python 3 (scikit-learn and SciPy).

## 3. Results

### 3.1. Study Population

A total of 46 women who underwent embryo transfer during the study period met the inclusion criteria and were included in the analysis. Biochemical pregnancy was achieved in 10 of 46 cycles (21.7%).

### 3.2. Baseline Characteristics

The baseline characteristics of the study population stratified by biochemical pregnancy outcome are presented in Table 5. The mean age of the study population was 33.1 ± 6.3 years (range: 23–46). Women who achieved biochemical pregnancy were significantly younger than those who did not (28.2 ± 4.2 vs. 34.4 ± 6.1 years; *p* = 0.001). Age was by far the strongest single baseline predictor, with age alone discriminating biochemical pregnancy with an AUC of 0.79. No significant differences were observed between groups for height, weight, menstrual cycle length, number of previous embryo transfers, or oocyte count (all *p* > 0.05).

None of the categorical baseline variables differed significantly between the biochemical pregnancy–positive and biochemical pregnancy–negative groups (all *p* > 0.05). These comprised polycystic ovaries, male factor infertility, endometriosis, low ovarian reserve, unexplained infertility, and term birth history, together with IVF cycle type (natural vs. hormonal), embryo status (fresh vs. frozen), embryo developmental stage (day 3 vs. day 5–6), and number of embryos transferred. The majority of cycles involved frozen embryo transfer (93.5%) and natural cycle preparation (54.3%).

### 3.3. Endometrial Parameters

Endometrial thickness measured on the day of embryo transfer was significantly greater in the biochemical pregnancy–positive group compared to the biochemical pregnancy–negative group (10.49 ± 1.54 vs. 9.05 ± 2.56 mm; *p* = 0.035, Welch’s *t*-test, used here because the group variances were unequal; the pooled-variance *t*-test gave *p* = 0.098). Endometrial volume showed a trend toward higher values in the biochemical pregnancy–positive group without reaching statistical significance (3.69 ± 1.30 vs. 2.78 ± 1.27 mL; *p* = 0.070). Mean uterine artery pulsatility index did not differ significantly between groups (2.78 ± 0.89 vs. 2.70 ± 0.74; *p* = 0.794).

Among categorical endometrial variables, endometrial morphology (pattern grading) showed the strongest association with pregnancy outcome (*p* = 0.005 before Bonferroni correction), though this did not retain significance after multiple comparison correction (adjusted *p* = 0.077).

Serial endometrial thickness measurements demonstrated progressive thickening from cycle baseline (4.68 ± 1.38 mm) through mid-cycle (7.73 ± 2.79 mm) to embryo transfer day (9.36 ± 2.44 mm).

### 3.4. Scoring System Performance

**Figure 2:**
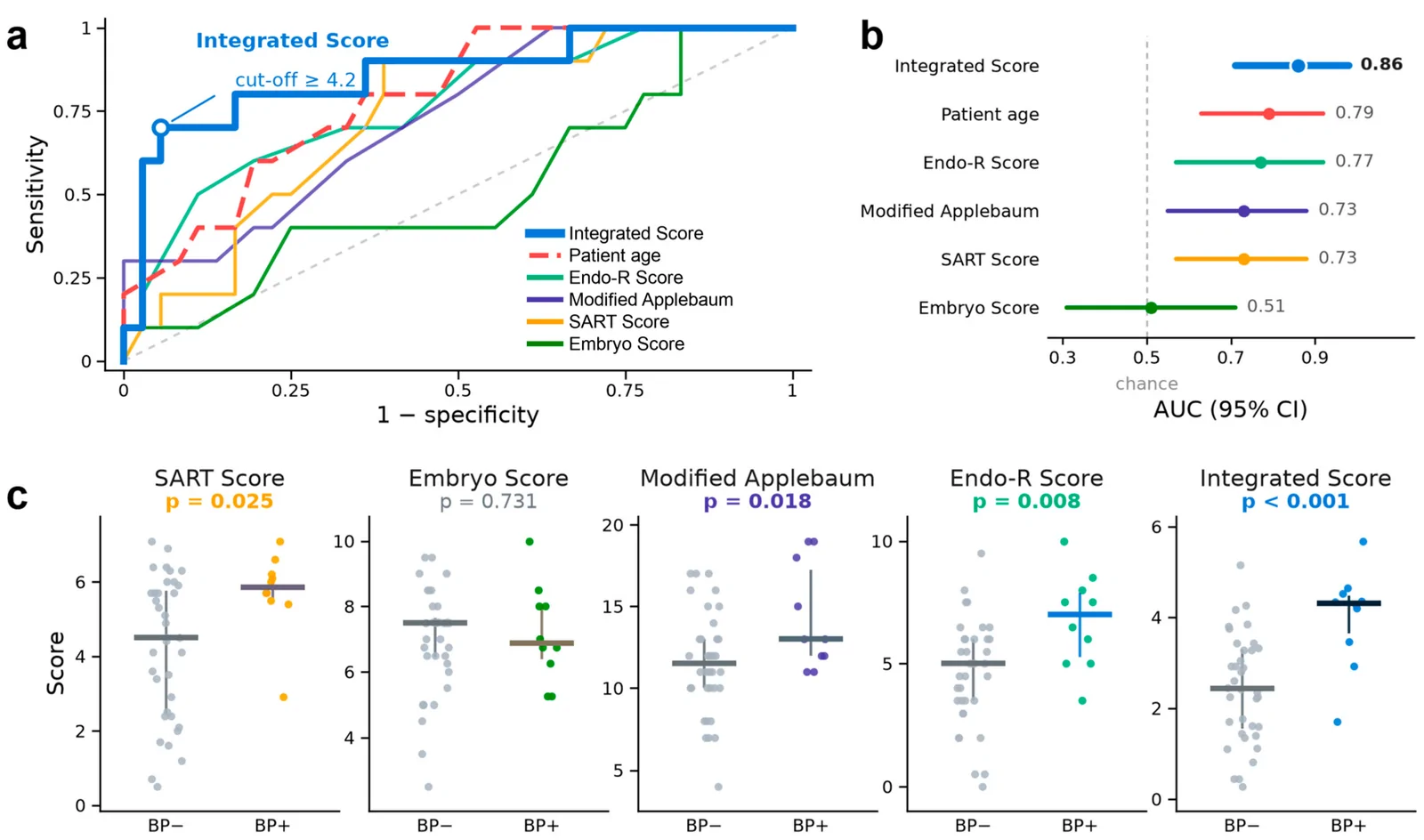
Discrimination of each scoring system for biochemical pregnancy (*n* = 46, 10 events). (**a**) Receiver operating characteristic curves; the Integrated Score (blue, heavy line) achieved the highest area under the curve, but the patient–age curve (red, dashed) tracks it closely. The diagonal denotes chance, and the open circle marks the Youden-optimal cut-off of the Integrated Score (≥4.2), which was derived and evaluated in the same cohort and is therefore optimistic. (**b**) Area under the curve with 95% bootstrap confidence intervals (2000 replicates); the intervals are wide and largely overlapping. Overlap of marginal intervals does not itself test a paired difference, which was assessed by bootstrap resampling (Section 3.4). (**c**) Distribution of each score by outcome; each point is one transfer cycle, horizontally jittered, with the horizontal bar marking the median and the vertical line the interquartile range. *p*-values are from independent samples *t*-tests and are uncorrected. The Embryo Score is the only component that does not separate the groups. Colors are consistent across all three panels. Abbreviations: AUC, area under the receiver operating characteristic curve; BP−, biochemical pregnancy negative; BP+, biochemical pregnancy positive; Endo-R, endometrial receptivity; SART, Society for Assisted Reproductive Technology.

The performance of each scoring system in predicting biochemical pregnancy is summarized in Table 6, with the corresponding receiver operating characteristic curves (Figure 2a), the areas under the curve and their confidence intervals (Figure 2b), and the score distributions by outcome (Figure 2c).

**SART Pregnancy Probability Score:** The mean SART Score was 4.57 ± 1.85 (range: 0.5–7.1). Scores were significantly higher in the biochemical pregnancy–positive group (5.72 ± 1.12 vs. 4.25 ± 1.90; t = 2.32, *p* = 0.025; AUC = 0.73).

**Embryo Morphological Quality Score:** The mean embryo score was 7.02 ± 1.56 (range: 2.5–10.0). No statistically significant difference was observed between groups (7.17 ± 1.48 vs. 6.98 ± 1.60; t = 0.35, *p* = 0.73; AUC = 0.51), consistent with the expectation that morphologically similar embryos were transferred across both groups.

**Modified Applebaum Score:** The mean Modified Applebaum Score was 12.13 ± 3.32 (range: 4–19). Scores were significantly higher in the biochemical pregnancy–positive group (14.30 ± 3.23 vs. 11.53 ± 3.12; t = 2.47, *p* = 0.018; AUC = 0.73). Pregnancy rates were 17.6% (6/34), 16.7% (1/6), and 50.0% (3/6) for Modified Applebaum scores of ≤13, 14–16, and ≥17, respectively, with only the highest stratum showing an elevated rate; these subgroup counts are very small and should be regarded as descriptive. The lowest stratum is nonetheless notable, because both the original report [10] and the frozen-transfer series [11] found no conceptions at scores of 13 or below, whereas 6 of our 34 such cycles conceived. A score of ≤13 therefore does not identify cycles that cannot conceive.

**Endo-R Score:** The mean Endo-R Score was 5.10 ± 2.26 (range: 0–10). Scores were significantly higher in the biochemical pregnancy–positive group (6.75 ± 1.93 vs. 4.64 ± 2.15; t = 2.80, *p* = 0.008; AUC = 0.77, 95% CI 0.57–0.92). The Endo-R Score achieved a numerically higher AUC than the seven-parameter Modified Applebaum Score (0.77 vs. 0.73), although this difference was not statistically significant (ΔAUC = 0.04, *p* = 0.56) and the two scores were strongly correlated (r = 0.77). Reciprocal likelihood-ratio tests showed that neither score added significant information to the other (Modified Applebaum added to a model containing Endo-R: χ^2^ = 0.16, df = 1, *p* = 0.69; Endo-R added to a model containing Modified Applebaum: χ^2^ = 2.22, df = 1, *p* = 0.14).

**Integrated Implantation Score: The mean Integrated Score was 2.78 ± 1.32 (observed range 0.29–5.68). The observed values occupied only the lower portion of the nominal 0–10 scale, because the patient-prognosis multiplier is typically well below 1, so the index compresses toward the lower end even when the embryo and endometrial components are favorable.** Scores were substantially higher in the biochemical pregnancy–positive group (4.01 ± 1.08 vs. 2.44 ± 1.18; t = 3.79, *p* = 0.0005), and the score achieved the highest apparent discrimination of any index evaluated (AUC = 0.86, 95% CI 0.71–0.98). Patient age alone achieved an AUC of 0.79 (95% CI 0.63–0.92); the difference between the two did not reach statistical significance (ΔAUC 0.072, 95% CI −0.07 to 0.23; *p* = 0.33). Across the five pairwise comparisons, the Integrated Score exceeded the Embryo Score by ΔAUC 0.350 (*p* = 0.0003; Bonferroni-corrected *p* = 0.0015), the SART Score by 0.129 (*p* = 0.024; corrected *p* = 0.12), and the Modified Applebaum Score by 0.131 (*p* = 0.117; corrected *p* = 0.59). The differences from the Endo-R Score (0.093; uncorrected *p* = 0.356) and from patient age (0.072; uncorrected *p* = 0.327) had Bonferroni-corrected *p*-values above unity (>1.0). Only the difference from the Embryo Score survives correction for these five comparisons.

On internal evaluation, the leave-one-out cross-validated AUC was 0.80 and the mean repeated 5-fold cross-validated AUC was 0.82. Fold-level variability was substantial (standard deviation across folds 0.16), reflecting approximately two events per fold; the previously reported value of 0.01 described only the dispersion of the averaged estimates across repetitions and understated the uncertainty of the estimate itself. Patient age alone achieved a leave-one-out cross-validated AUC of 0.73.

Two sensitivity analyses were performed (Table S3). Excluding the four cycles with imputed embryo scores (*n* = 42, 10 events), the Integrated Score achieved an AUC of 0.869 (t = 3.84, *p* = 0.0004), with Endo-R 0.761, SART 0.733, patient age 0.805, and Embryo 0.525. Restricting the cohort to the 35 cycles with complete, stage-appropriate embryo grading (8 events; this excludes both the 7 summary-graded day-5 cycles and the 4 cycles with imputed sub-grades, which did not overlap) gave an Integrated Score AUC of 0.847, Endo-R 0.773, patient age 0.843, and Embryo 0.375. Neither analysis changed the direction or significance of any comparison, and in both the Integrated Score remained close to patient age alone. The Embryo Score itself was unstable across these subsets (0.525 and 0.375 against 0.51 in the full cohort), which at eight to ten events is uninformative in either direction and is consistent with the absence of any measurable contribution from this component. Two ways of neutralizing the embryo term were examined, and neither changed discrimination materially. Replacing the term with a constant gave an AUC of 0.856 versus 0.861 for the pre-specified formula (ΔAUC = 0.006 on unrounded values, *p* = 0.97), while dropping it altogether, in the two-domain variant (SART/10) × Endo-R, gave 0.858 (ΔAUC = 0.003; Supplementary Table S2).

At the Youden-optimal cut-off (≥4.2), the Integrated Score classified biochemical pregnancy with a sensitivity of 70.0%, specificity of 94.4%, positive predictive value of 77.8%, negative predictive value of 91.9%, and accuracy of 89.1%. Because this threshold was both derived and evaluated in the same 46 cycles, these operating characteristics are optimistic and require external confirmation before any use. This cut-off–based accuracy of 89.1% (41/46) differs from the 82.6% logistic-model classification accuracy in Table 7, which reflects a different classification rule applied to the same data. A threshold intended for counseling must be fixed in a derivation cohort and tested in an independent one before it carries any clinical meaning.

### 3.5. Logistic Regression Analysis

Logistic regression models were constructed to evaluate the independent predictive value of the scoring components (Table 7).

Model 1 (SART Score + Embryo Score + Modified Applebaum Score): apparent AUC = 0.86, cross-validated AUC = 0.76, likelihood-ratio χ^2^ = 13.64, df = 3, *p* = 0.003. Individual coefficients: SART *p* = 0.03, Modified Applebaum *p* = 0.02, Embryo *p* = 0.84.

Model 2 (SART Score + Embryo Score + Endo-R Score): apparent AUC = 0.85, cross-validated AUC = 0.76, likelihood-ratio χ^2^ = 15.07, df = 3, *p* = 0.002. Individual coefficients: SART *p* = 0.03, Endo-R *p* = 0.015, Embryo *p* = 0.79.

Model 3 (Integrated Score alone): apparent AUC = 0.86, cross-validated AUC = 0.80, likelihood-ratio χ^2^ = 13.18, df = 1, *p* = 0.0003.

On internal validation, the single-variable Integrated Score model (Model 3, cross-validated AUC = 0.80) showed a numerically higher cross-validated AUC than the multivariable models combining the three individual domains (cross-validated AUC = 0.76 for both). With only 10 events, however, a difference of this magnitude is well within the range attributable to noise, and no claim of superior internal validity is made for the pre-specified single-variable index over a fitted multivariable model. The individual coefficients are informative: in Model 2, the SART (*p* = 0.03) and Endo-R (*p* = 0.015) terms were significant while the Embryo term was not (*p* = 0.79), and the same pattern held in Model 1 (SART *p* = 0.03, Modified Applebaum *p* = 0.02, Embryo *p* = 0.84).

Because paired AUC comparisons have low power at this event count, the incremental contribution of each component beyond patient age was additionally examined by likelihood-ratio tests on nested logistic models. Adding the Endo-R Score to a model containing age improved fit (χ^2^ = 8.70, df = 1, *p* = 0.003), as did adding the Integrated Score (χ^2^ = 5.94, *p* = 0.015), whereas adding the Embryo Score did not (χ^2^ = 0.42, *p* = 0.52). The Endo-R Score also added information beyond the SART Score (χ^2^ = 8.86, *p* = 0.003). These tests were not pre-specified, rest on 10 events, and are reported as exploratory; they indicate that the endometrial domain carries information not contained in age alone, but they cannot establish clinical incremental value.

### 3.6. Correlation Analysis

Inter-score correlations clarified the relationships among the components. The Integrated Score was strongly correlated with the SART Score (r = 0.86) and inversely with patient age (r = −0.64), quantifying the extent to which the composite reflects patient prognosis. The Endo-R Score and Modified Applebaum Score were strongly correlated (r = 0.77), as expected given their overlapping endometrial parameters, indicating that these two endometrial scores are not independent comparators. In contrast, the Embryo Score was essentially uncorrelated with both endometrial scoring systems (|r| ≤ 0.04), confirming that the embryo and endometrial assessment domains capture independent information.

## 4. Discussion

This proof-of-concept pilot study developed and internally evaluated an Integrated Implantation Score combining patient prognosis, embryo morphological quality, and endometrial receptivity to predict biochemical pregnancy following embryo transfer in IVF. The central result is negative with respect to the study’s motivating hypothesis: although the Integrated Score achieved the numerically highest discrimination of all indices examined (apparent AUC 0.86; cross-validated 0.80), it did not significantly outperform patient age alone (AUC 0.79; *p* = 0.33), and its embryo component contributed no discrimination (AUC 0.51). The sections that follow examine where the predictive signal resides, what the individual components contribute, and what the study delivers for the work that must follow.

### 4.1. Patient Age as a Dominant Predictor and Potential Confounder

The dominant observation is that patient age alone discriminated biochemical pregnancy nearly as well as the full Integrated Score (AUC 0.79 vs. 0.86; ΔAUC 0.072, *p* = 0.33). Age is also a principal input to the SART prognosis model, which in turn scales the Integrated Score, so the composite is correlated with the SART Score at r = 0.86 and with age at r = −0.64: a substantial part of its apparent performance necessarily restates the well-established effect of age on IVF success. Recent large-cohort data reinforce how strong this effect is [5]. Exploratory likelihood-ratio tests offer a partial qualification. The endometrial component improved model fit beyond age (Endo-R Score, χ^2^ = 8.70, *p* = 0.003) and beyond the SART Score (χ^2^ = 8.86, *p* = 0.003), whereas the embryo component did not (χ^2^ = 0.42, *p* = 0.52). This suggests that if the score carries information beyond age, that information resides in the ultrasound domain rather than the embryo domain. We stress that these tests were not pre-specified, rest on 10 events, and are themselves fragile; they refine the negative headline finding but do not overturn it. Whether the Integrated Score offers genuine incremental value over age and the SART Score remains the single most important question to resolve, and the present results should not be interpreted as demonstrating such value. One design implication follows directly. Because age carries so much of the signal here, the most efficient test of whether the score adds anything is a cohort in which age itself varies little. Within a narrow age band, for example women aged 30–35 years, age discriminates weakly, so any incremental contribution of the endometrial and embryo domains would be directly visible. An age-stratified or age-restricted validation cohort is therefore arguably the more informative design, and we identify this as a central design question for the next study.

### 4.2. The Rationale for an Integrated Approach

It is important to separate the statistical result from the biological reasoning here, because the two point in different directions. The statistical result is unambiguous: the Embryo Score did not differ between outcome groups (7.17 ± 1.48 vs. 6.98 ± 1.60; *p* = 0.73), did not discriminate (AUC 0.51), added nothing to a model containing age, and its removal from the formula left discrimination unchanged. By any measure available in this dataset, the embryo component contributed nothing. The biological argument for retaining it is separate and is not supported by these data. A viable embryo is a necessary condition for implantation, and the absence of a measurable contribution here most plausibly reflects the restricted range of embryo quality in this cohort, from which only morphologically favorable embryos were transferred. Yang et al. reported a related observation in cycles using at least one morphologically good-quality blastocyst: endometrial thickness and pattern were associated with clinical pregnancy, although neither predicted live birth, and those authors concluded that embryo quality was the more consequential factor [25]. Their finding supports the narrower claim that endometrial assessment retains information when embryo quality is uniformly favorable; it does not support any inference that embryo quality is unimportant. We therefore retained the embryo term as a pre-specified structural element rather than a demonstrated discriminator, since removing it would tailor the index to the embryo-quality distribution of this cohort, itself a form of overfitting. The three-domain structure should be read as a hypothesis carried forward on biological grounds, not as a structure validated here; testing it requires a cohort with wider variation in embryo quality.

Within the endometrial domain, the question is a different one: not whether it contributes, but how much measurement that contribution requires. The Endo-R Score achieved a numerically higher AUC than the more complex Modified Applebaum Score (0.77 vs. 0.73) despite using five parameters rather than seven, although this difference was not statistically significant (ΔAUC = 0.04, *p* = 0.56). The two scores overlap substantially by construction. Four of the five Endo-R parameters (endometrial thickness, endometrial pattern, zone 3 endometrial blood flow, and mean uterine artery pulsatility index) also appear in the Modified Applebaum system, and the two scores are correlated at r = 0.77. They are therefore not independent comparators, and a formal comparison between them has less power than the event count would suggest. The Modified Applebaum system additionally includes three myometrial and uterine-artery parameters that Endo-R omits (myometrial echogenicity, uterine artery end-diastolic flow, and myometrial blood flow); omitting these did not reduce discrimination in this cohort, but with 46 cycles we cannot determine whether they contribute to larger samples or other populations. The practical implication is therefore one of measurement economy rather than performance: a five-parameter assessment obtainable in a single transvaginal examination on the morning of transfer appears sufficient here to capture what the seven-parameter instrument measures, which may lower the barrier to routine use. Neither equivalence nor superiority is established by these data; demonstrating either would require a head-to-head comparison in an independent cohort powered for the small differences involved.

A final structural question concerns not the components themselves but the rule that combines them. The multiplicative integration of patient prognosis with the embryo–endometrium assessment reflects the clinical reasoning that optimal local conditions cannot fully compensate for unfavorable patient prognosis, and vice versa. We tested this structural choice post hoc against five alternatives (Supplementary Materials). The apparent AUCs ranged from 0.761 to 0.861, but these values cannot be ranked meaningfully. With 10 events and 36 non-events, the AUC is estimated over 360 case–control pairs and can change only in steps of 1/360 (0.003); the six structures span 274 to 310 such pairs. The three highest-scoring formulations lie within six pairs of one another, and the two variants that neutralize the embryo term are within one to two pairs of the pre-specified formula (0.858 and 0.856 versus 0.861), which is the resolution limit of this dataset. No pairwise difference approached significance. The only defensible conclusion is negative: these data cannot distinguish between candidate formulations, so the pre-specified formula is not shown to be correct, merely not contradicted. The formula structure is accordingly the first target of the planned derivation study, and the near-identical performance of the two-domain variant is consistent with the absent contribution of the embryo component discussed above.

### 4.3. Comparison with Previous Studies

Zhang et al. [26] proposed an endometrial receptivity scoring system based on endometrial thickness, volume, echo pattern, peristalsis, and blood flow evaluated by ultrasonography. Comprehensive though it is, their system incorporates neither embryo quality nor patient prognostic factors. Sapkota and Sharma [11] likewise applied a Modified Applebaum scoring system in 200 frozen embryo transfer cycles. They reported pregnancy rates of 97.4% at a perfect score of 20, 85.4% at 17–19, and 28.6% at 14–16, with no pregnancies at scores of 13 or below. The original profile describes the same pattern, including the same lower boundary [10]. Our cohort reproduces the direction of that gradient but neither its steepness nor its lower bound, since 17.6% of cycles scoring ≤13 conceived. The score distribution here is also less favorable (mean 12.13 ± 3.32), which points to a difference in case mix rather than in scoring. Two further differences plausibly contribute: our outcome is biochemical pregnancy in a small single-center sample, and the published bands were preliminary. The specificity of 0.59 reported by Sapkota and Sharma nonetheless indicates the same limitation we observe, that a high score is common among cycles that do not conceive. Our findings agree with theirs on the predictive value of uterine biophysical parameters, and extend the approach by integrating embryo and patient-level data.

The concept that endometrial receptivity markers alone have high sensitivity but low specificity has been well established [8]. Endometrial thickness, although associated with outcome in large series [27], is an imperfect discriminator on its own; earlier work combining spiral artery flow, thickness, volume and uterine artery flow reached the same conclusion for multiparametric endometrial assessment [28]. At its Youden-derived cut-off, the Integrated Score achieved a specificity of 94% with a sensitivity of 70%. That threshold was selected in the same cohort in which it was evaluated, however, and the composite did not outperform patient age, so these operating characteristics cannot be read as evidence that combining domains resolves the specificity problem. Notably, our deliberately simple, ultrasound-based composite is intended to complement rather than replace molecular approaches to endometrial receptivity, such as endometrial receptivity array–guided personalized embryo transfer, whose clinical benefit remains debated [29]. Not every component of the endometrial score earned its place, however. Mean uterine artery pulsatility index did not differ between outcome groups (*p* = 0.79) and most patients fell into a single scoring category. On the present evidence, this component is a candidate for removal from a future version of the score, and the derivation study should test the four-parameter Endo-R Score against the five-parameter version directly.

### 4.4. Clinical Implications

The present data do not support any change in clinical management, and do not justify withholding or canceling an embryo transfer based on the score. Should adequately powered, externally validated work confirm incremental value beyond patient age for clinical pregnancy and live birth, the index could serve as a standardized research instrument for quantifying the combined patient–embryo–endometrial milieu, or to flag cycles for closer study of endometrial preparation. Its practical attraction is simplicity: routine ultrasound parameters, standard embryo grading, and readily available patient data, with no specialized equipment or additional invasive procedures.

### 4.5. Limitations

This study has several important limitations. The principal one is its size. With 46 cycles and 10 positive outcomes, statistical power is low, and the sample falls well below what formal criteria recommend for developing a multivariable prediction model [30]. At this event count, only very large differences between correlated AUCs are detectable, which is why the comparison against age should be read as inconclusive rather than as evidence of equivalence; the low event rate also precluded multivariable adjustment for confounders and meaningful subgroup analyses. The study was conducted at a single center, introducing potential selection and measurement bias. The choice of endpoint compounds these constraints: biochemical pregnancy is a proximal surrogate that includes pregnancies which do not progress, and discrimination for it does not establish discrimination for clinical pregnancy or live birth, the endpoints that matter to patients. Relatedly, the SART-based prognosis model was applied outside its validated endpoint (cumulative live birth) and its derivation population (United States registry data), so its calibration to single-transfer biochemical pregnancy in a Turkish cohort is uncertain, and it was used to rank patients rather than to provide an absolute probability. Because its principal inputs are age and ovarian reserve [15], the ordering it produces is expected to be largely preserved across endpoints even where absolute calibration does not transfer.

The index itself rests on choices these data cannot validate. The formula was pre-specified on clinical grounds and remains a hypothesis rather than a validated structure; internal cross-validation tempered in-sample optimism, but cross-validated estimates obtained within a single small cohort still tend to overestimate true external performance, and fold-level variability was substantial (standard deviation across folds 0.16). We assessed discrimination only, because the low event count would render formal calibration estimates unstable, and calibration must be established before any individualized probability is reported. The embryo component carries a further assumption: established Baczkowski and Gardner grades were mapped onto a common 0–10 scale that has not been independently validated, and the equivalence of cleavage- and blastocyst-stage scores is assumed rather than established. With 10 events divided across two developmental stages, a stage-stratified evaluation of the embryo component would not be interpretable in this cohort and was not attempted. In seven cycles, a day-5 embryo was scored using cleavage-stage parameters because only a summary laboratory grade was recorded, although a sensitivity analysis restricted to stage-appropriately graded cycles did not alter the findings, and the median imputation of four embryo scores is unlikely to have affected the results, as the complete-case analysis confirmed. The assessment was also based on static morphology; time-lapse morphokinetic parameters [31] or preimplantation genetic testing could enhance this component. The Endo-R cut-offs were literature-derived rather than cohort-optimized, which may have limited discrimination; conversely, the Youden-derived threshold for the Integrated Score was selected in the same sample in which it was evaluated and is therefore optimistic.

Features of the cohort and of the measurement process further restrict what can be generalized. The cohort was overwhelmingly frozen-transfer (93.5%, 43/46), so fresh and frozen cycles could not be meaningfully compared, and the results apply essentially to frozen transfers; endometrial preparation was split between natural (54.3%) and hormonally prepared (45.7%) cycles, which differ in the hormonal milieu governing the very parameters the Endo-R Score measures. Neither transfer type nor cycle type differed significantly between outcome groups, but the event count precluded stratified analysis, leaving both as unresolved sources of confounding; a validation cohort should either stratify by endometrial-preparation protocol or restrict to a single protocol. All ultrasound assessments were performed and scored by a single operator, which eliminates inter-observer variability within the study but makes the results conditional on one observer, so the reported discrimination is an upper bound on what would be achieved in multi-operator practice. Endometrial pattern and zone 3 vascularity are the most vulnerable components, and a formal reproducibility substudy, reporting the intraclass correlation coefficient for continuous components and Cohen’s kappa for categorical ones, is required; the Doppler settings held constant here are themselves a source of between-center variability. Outcomes were ascertained retrospectively from clinical records; because all scoring inputs were recorded prospectively and before the outcome was known, outcome-driven bias is unlikely, but ascertainment was not blinded by design. Double-embryo transfer was performed in 56.5% of cycles, for which only the higher-scoring embryo contributed to the embryo component; this discards information from the co-transferred embryo and ignores the higher per-cycle implantation probability that two embryos confer independently of morphology. The score does not model transfer number at all; a future version should either include it as an explicit term or restrict to single-embryo transfers, the cleaner design for a derivation study.

### 4.6. Future Directions

Beyond its own result, the study is intended to lower the practical cost of the multicenter work it calls for. Standardizing receptivity assessment across centers is itself a recognized obstacle: the parameters are operator and machine-dependent, and published scoring systems differ in which parameters they include and how they are banded. Every rule used here is therefore reported in full (Table 1, Table 2, Table 3 and Table 4), the Endo-R thresholds are literature-derived rather than fitted to this cohort so that they can be applied unchanged elsewhere, and the index is implemented as a browser-based calculator with source code released under an open license [22]. The pre-defined primary question, whether endometrial assessment adds to patient age for clinical pregnancy and live birth, together with the effect sizes reported here, provides the basis for powering such a study. Concretely, that study should be a multicenter prospective cohort with a pre-registered protocol and analysis plan, powered for the incremental-value question against age rather than for overall discrimination. It should be stratified by, or restricted to, a single endometrial-preparation protocol, and should incorporate a formal inter-observer reproducibility substudy reporting the intraclass correlation coefficient or kappa for each component, using clinical pregnancy and live birth as primary endpoints, and reporting calibration alongside discrimination. The score should be re-derived rather than merely re-applied if a validation cohort differs materially in age distribution from the present one.

Methodological refinement will be central to that work. The embryo component should be re-examined in a cohort with wider morphological variation, with its predictive value evaluated separately for cleavage and blastocyst-stage embryos, and the endometrial component enriched with more advanced ultrasound techniques, namely real-time endometrial strain elastosonography and three-dimensional vascularization indices [19], which have shown promise for receptivity assessment in pilot work [32]. The role of artificial intelligence and machine-learning algorithms in automating endometrial and embryo assessment and potentially enhancing the predictive accuracy of integrated scoring systems is a further avenue for investigation [33].

## 5. Conclusions

This proof-of-concept pilot study integrated patient prognosis, embryo morphological quality and endometrial receptivity into a single transparent index and evaluated it internally. The primary result is negative: the Integrated Score achieved the highest apparent discrimination of the indices examined (AUC 0.86) but did not significantly surpass patient age alone (0.79), and its embryo component contributed no discrimination. The endometrial component, by contrast, carried information independent of both age and the patient-prognosis model, identifying it as the domain where an integrated index is most likely to earn its place.

The study’s contribution is therefore methodological rather than clinical. It provides a fully specified scoring rubric, an openly available calculator with published source code and a primary question defined in advance for the study that must follow whether endometrial assessment adds to patient age in predicting clinical pregnancy and live birth. These materials are intended to make a multicenter derivation and validation study directly executable, by supplying a common scoring language, comparable measurement across centers, and a reproducible implementation. That study, not the present one, is where the question can be answered.

## Figures and Tables

**Table 1:** Cleavage-stage embryo morphological quality score (Day 3).

Parameter	Criteria	Score
Cell number	Normal range (6–10 cells)	2
	Below normal range	0
Fragmentation	Grade 1 (≤10%)	3
	Grade 1–2 (10–20%)	2.5
	Grade 2 (20–35%)	2
	Grade 2–3 (35–50%)	1.5
	Grade 3 (>50%)	1
Symmetry	Symmetrical/Fairly symmetrical	3
	Minor asymmetry	2
	Moderate asymmetry	1
Cytoplasm	Uniform/Homogeneous	1
	Minor granularity	0.5
	Granular/Irregular	0
Zona pellucida	Visible and intact	1
	Irregular/Thin	0

Maximum score: 10 points. Cleavage-stage embryos were graded on the five Baczkowski morphological parameters listed; points increase with grade quality and are summed. Where a parameter was recorded as a range (for example fragmentation grade 1–2), the midpoint of the corresponding point values was used.

**Table 2:** Blastocyst-stage embryo morphological quality score (Day 5–6).

Parameter	Criteria	Score
Expansion	Grade 5–6 (hatching/hatched)	3
	Grade 4 (expanded)	2.5
	Grade 3 (full)	2
	Grade 2 (early)	1
	Grade 1 (cavitating)	0.5
ICM quality	Grade A (tightly packed)	3.5
	Grade B (loosely grouped)	2
	Grade C (few/disorganized)	0.5
TE quality	Grade A (many cells, cohesive)	3.5
	Grade B (few cells, loose)	2
	Grade C (very few, large)	0.5

Maximum score: 10 points. Blastocysts were graded on the three Gardner parameters listed; points increase with grade quality and are summed. Where a parameter was recorded as a range (for example expansion grade 5–6), the mean of the corresponding point values was used. Abbreviations: ICM, inner cell mass; TE, trophectoderm.

**Table 3:** Modified Applebaum uterine scoring system.

Parameter	Criteria	Score
Endometrial thickness (mm)	<7	0
	7–9	2
	10–14	3
	>14	1
Endometrial morphology	No layering	0
	Blurred five-line	1
	Clear five-line	3
Myometrial echogenicity	Coarse/Heterogeneous	1
	Fairly homogeneous	2
Uterine artery end-diastolic flow	Absent/Reversed	0
	Present	3
Uterine artery Doppler PI	≥2.5	0
	2.2–2.49	1
	≤2.19	2
Zone 3 endometrial blood flow	Absent	0
	Present, sparse	2
	Abundant, multifocal	5
Myometrial blood flow	Absent (minimal)	0
	Present	2

Maximum score: 20 points. The thickness score is non-monotonic: values above 14 mm score lower than the 10–14 mm band, as in the original Applebaum profile. Point values follow that profile, except that its two lowest pulsatility index bands, which both score 0, are combined here as ≥2.5. Abbreviations: PI, pulsatility index.

**Table 4:** Endometrial receptivity score (Endo-R Score).

Parameter	Criteria	Score
Endometrial thickness	7–14 mm	2
	<7 mm or >14 mm	0
Endometrial pattern	Trilaminar, clear, uniform	2
	Non-trilaminar	0
Endometrial volume	≥2 mL	2
	<2 mL	0
Zone 3 endometrial blood flow	Abundant, multifocal	2
	Present, sparse	1
	Absent	0
Mean uterine artery PI	≤2.19	2
	2.20–2.49	1
	≥2.50	0

Maximum score: 10 points. The thickness component is non-monotonic by design: both <7 mm and >14 mm score zero, because excessive thickness is not regarded as reassuring. A trilaminar pattern was defined as a symmetrical three-line appearance with a distinct central echogenic line and uniform hypoechoic outer layers; any other appearance was scored as non-trilaminar. Abbreviations: Endo-R, endometrial receptivity; PI, pulsatility index.

**Table 5:** Baseline characteristics by pregnancy outcome.

Characteristic	BP-Positive (*n* = 10)	BP-Negative (*n* = 36)	*p*
Age (years)	28.2 ± 4.2	34.4 ± 6.1	0.001
Height (cm)	163.9 ± 4.6	163.1 ± 4.0	0.59
Weight (kg)	64.7 ± 9.2	69.1 ± 13.3	0.33
Menstrual cycle length (days)	29.7 ± 3.4	29.6 ± 3.7	0.95
No. of previous embryo transfers	0.6 ± 1.0	0.9 ± 1.0	0.47
Oocyte number	13.2 ± 7.0	9.5 ± 5.3	0.078
Endometrial thickness on ET day (mm)	10.49 ± 1.54	9.05 ± 2.56	0.035

Values are mean ± SD. *p*-values from the independent samples *t*-test, with Welch’s correction applied where the group variances were unequal. Abbreviations: BP, biochemical pregnancy; ET, embryo transfer; SD, standard deviation.

**Table 6:** Comparison of scoring systems.

Scoring System	Scale	Mean ± SD	BP-Positive (*n* = 10)	BP-Negative (*n* = 36)	t	*p*	AUC (95% CI)
SART Score	0–10	4.57 ± 1.85	5.72 ± 1.12	4.25 ± 1.90	2.32	0.025	0.73 (0.57–0.88)
Embryo Score	0–10	7.02 ± 1.56	7.17 ± 1.48	6.98 ± 1.60	0.35	0.73	0.51 (0.31–0.71)
Modified Applebaum Score	0–20	12.13 ± 3.32	14.30 ± 3.23	11.53 ± 3.12	2.47	0.018	0.73 (0.55–0.88)
Endo-R Score	0–10	5.10 ± 2.26	6.75 ± 1.93	4.64 ± 2.15	2.80	0.008	0.77 (0.57–0.92)
Integrated Score	0–10	2.78 ± 1.32	4.01 ± 1.08	2.44 ± 1.18	3.79	0.0005	0.86 (0.71–0.98)

Scale denotes the theoretical range of each score; observed ranges are given in the text. AUC values are apparent (in-sample) with 95% bootstrap confidence intervals (2000 replicates). Group comparisons used the pooled-variance independent samples *t*-test throughout; Welch statistics for the unequal-variance comparisons are reported in Section 3.3. For the Integrated Score, the cross-validated AUC was 0.80 (leave-one-out) and 0.82 (repeated 5-fold; standard deviation across folds 0.16). Pairwise AUC differences, with uncorrected and Bonferroni-corrected *p*-values, are reported in Section 3.4; they were assessed by bootstrap resampling with 4000 replicates. Abbreviations: AUC, area under the receiver operating characteristic curve; BP, biochemical pregnancy; CI, confidence interval; Endo-R, endometrial receptivity; SART, Society for Assisted Reproductive Technology; SD, standard deviation.

**Table 7:** Logistic regression models.

Model	Variables	Accuracy	Apparent AUC	CV-AUC	*p*
1	SART + Embryo + Modified Applebaum	84.8%	0.86	0.76	0.003
2	SART + Embryo + Endo-R Score	87.0%	0.85	0.76	0.002
3	Integrated Score	82.6%	0.86	0.80	0.0003

Accuracy and apparent AUC are in-sample (optimistic) estimates. *p*-values are likelihood-ratio tests of each model against the null model; the χ^2^ statistics, degrees of freedom and individual coefficient *p*-values are detailed in the text above. Models 1 and 2 are illustrative rather than definitive: with 10 events, three-predictor models are substantially over-parameterized, and their coefficients should not be interpreted individually. Abbreviations: AUC, area under the receiver operating characteristic curve; CV, cross-validated; Endo-R, endometrial receptivity; SART, Society for Assisted Reproductive Technology.

## Data Availability

The data presented in this study are available from the corresponding author upon reasonable request. The prototype calculator and its full source code are openly available at https://github.com/egehanbilen/endo-r-score (accessed on 20 June 2026).

## Supplementary Materials

The following supporting information can be downloaded at: https://www.mdpi.com/article/10.3390/diagnostics16162508/s1, Table S1: TRIPOD checklist; Table S2: Post-hoc comparison of alternative formula structures; Table S3: Sensitivity analyses; Figure S1: Prototype calculator; Supplementary Methods S1: Extended rationale for the embryo score point allocations and full description of the calculator implementation.
