# Supplementary material for: Development and Internal Evaluation of an Integrated Patient–Embryo–Endometrial Scoring System for Predicting Biochemical Pregnancy in IVF: A Proof-of-Concept Pilot Study
Source: Diagnostics (Basel). 2026 Aug 9;16(16):2508. doi: 10.3390/diagnostics16162508 (PMC13511637; doi:10.3390/diagnostics16162508)
Supplement: Supplementary file 1 [file diagnostics-16-02508-s001.zip › diagnostics-4443224-supplementary.pdf]

Supplementary Materials

Development and Internal Evaluation of an Integrated Patient–Embryo–Endometrial Scoring System for Predicting Biochemical Pregnancy in IVF: A Proof-of-Concept Pilot Study

Table S1. TRIPOD checklist

Completed against the TRIPOD statement (reference 23) and its updated guidance, TRIPOD+AI (reference 24). All 37 TRIPOD sub-items are listed. Sub-items covered jointly by a single manuscript location are grouped in one row; five sub-items are marked NOT APPLICABLE, each with a stated reason, because this study reports model development without a validation data set, and item 13c is likewise not applicable for that reason. Abbreviations: AUC, area under the receiver operating characteristic curve; Endo-R, endometrial receptivity; SART, Society for Assisted Reproductive Technology; TRIPOD, Transparent Reporting of a multivariable prediction model for Individual Prognosis or Diagnosis.

TRIPOD item	Description	Location in manuscript
1. Title	Identifies study as developing a prediction model, the target population and outcome	Title
2. Abstract	Structured summary of objectives, methods, results, conclusions	Abstract
3a. Background	Rationale, including reference to existing models	1. Introduction
3b. Objectives	Study objectives, specifying development vs validation	1. Introduction, final paragraph
4a. Source of data	Study design and rationale	2.1
4b. Key study dates	Start and end of accrual; follow-up end if applicable	2.1 (accrual March–December 2023); outcome assessed ~12 days after transfer (2.4)
5a-5c. Participants	Setting, eligibility, treatment received	2.1, 2.1.1, 2.1.2
6a-6b. Outcome	Definition, assessment, blinding to predictors	2.4; assessors described in 2.1
7a-7b. Predictors	Definition, assessment, blinding to outcome	2.2, 2.3.1–2.3.5
8. Sample size	How study size was arrived at	2.1 and 4.5 (convenience sample; limitation stated)
9. Missing data	How handled	2.3.2 (median imputation, n = 4); complete-case analysis in 3.4
10a-10d. Statistical analysis methods	Model development, performance measures, internal validation	2.5
10e. Model updating	Any model updating arising from validation	NOT APPLICABLE — no validation data set, so no updating performed
11. Risk groups	How defined, if done	3.4 (Youden threshold; stated as apparent)

12. Development vs. validation	Differences from development data in setting, eligibility, outcome, predictors	NOT APPLICABLE — development only; no validation data set
13a-13c. Participants (results)	Flow of participants, characteristics, comparison with development data	13a: 3.1; 13b: 3.2 and Table 5 (missing-predictor counts in 2.3.2 and 3.4); 13c not applicable (no validation data set)
14a. Model development (results)	Number of participants and outcome events in each analysis	3.1 (46 cycles, 10 events); per-analysis n and events in 3.4, 3.5, Tables 6 and 7; sensitivity analyses in 3.4 (42 and 35 cycles)
14b. Unadjusted associations	Unadjusted association between each candidate predictor and outcome, if done	3.2 and Table 5 (baseline variables); 3.3 and Table 6 (each score vs. outcome)
15a. Model specification	Full prediction model presented	2.3.1–2.3.5, Tables 1–4; open-source calculator
15b. Using the model	How to use the prediction model	2.3.5 and Equation; scoring rubric in Tables 1–4; open-source browser calculator described at the end of 2.3.5 (Figure S1)
16. Model performance	Discrimination with confidence intervals	3.4, Table 6, Figure 2
16 (calibration). Model performance — calibration	Calibration slope or plot	NOT APPLICABLE — not undertaken; 10 events render calibration estimates unstable (stated in 2.5 and 4.5)
17. Model updating (results)	Results of any model updating	NOT APPLICABLE — no updating performed

Table S2. Post-hoc comparison of alternative formula structures

These comparisons were performed after the primary analysis, were not used for model selection, and are reported to demonstrate that the present data cannot distinguish between candidate structures.

Formula	AUC (95% CI)
Two-domain: (S/10) × R	0.858 (0.71–0.97)
Pre-specified: (S/10) × (E + R)/2	0.861 (0.71–0.98)
Fully multiplicative: (S/10) (E/10) (R/10) × 10	0.844 (0.68–0.98)
Geometric mean of the three terms	0.844 (0.68–0.98)
Additive: (S + E + R)/3	0.819 (0.64–0.96)
Without prognosis term: (E + R)/2	0.761 (0.55–0.93)

S, SART Score; E, Embryo Score; R, Endo-R Score. Abbreviations: AUC, area under the receiver operating characteristic curve; CI, confidence interval; Endo-R, endometrial receptivity; SART, Society for Assisted Reproductive Technology.

No pairwise difference among these structures approached statistical significance. With 10 events and 36 non-events the AUC is estimated over 360 case–control pairs and can

change only in steps of 1/360 (0.003); every value in this table is an exact multiple of that step, and the six structures span 274 to 310 such pairs. The three highest-scoring formulations lie within six pairs of one another and cannot be meaningfully ranked at this event count. The two-domain variant, which omits the embryo term, is within one to two pairs of the pre-specified three-domain formula and so performed essentially identically to it, consistent with the absence of any measurable contribution from the embryo component in this cohort (see Section 4.2).

Table S3. Sensitivity analyses

**Analysis**	**n (events)**	**Integrated**	**Endo-R**	**Age**	Embryo
Full cohort	46 (10)	0.861	0.768	0.789	0.511
Complete cases (imputed embryo scores excluded)	42 (10)	0.869	0.761	0.805	0.525
Stage-appropriate embryo grading only	35 (8)	0.847	0.773	0.843	0.375

Values are apparent areas under the receiver operating characteristic curve. All four cycles with imputed embryo scores were biochemical pregnancy negative. Full-cohort values are shown to three decimals here for comparability with the sensitivity analyses; they are reported to two decimals in Table 6 of the main text. The Embryo Score varies across subsets (0.511, 0.525, 0.375) in a way that is uninformative at eight to ten events and consistent with the absence of any measurable contribution from this component. Abbreviations: AUC, area under the receiver operating characteristic curve; Endo-R, endometrial receptivity.

Figure S1. Prototype calculator

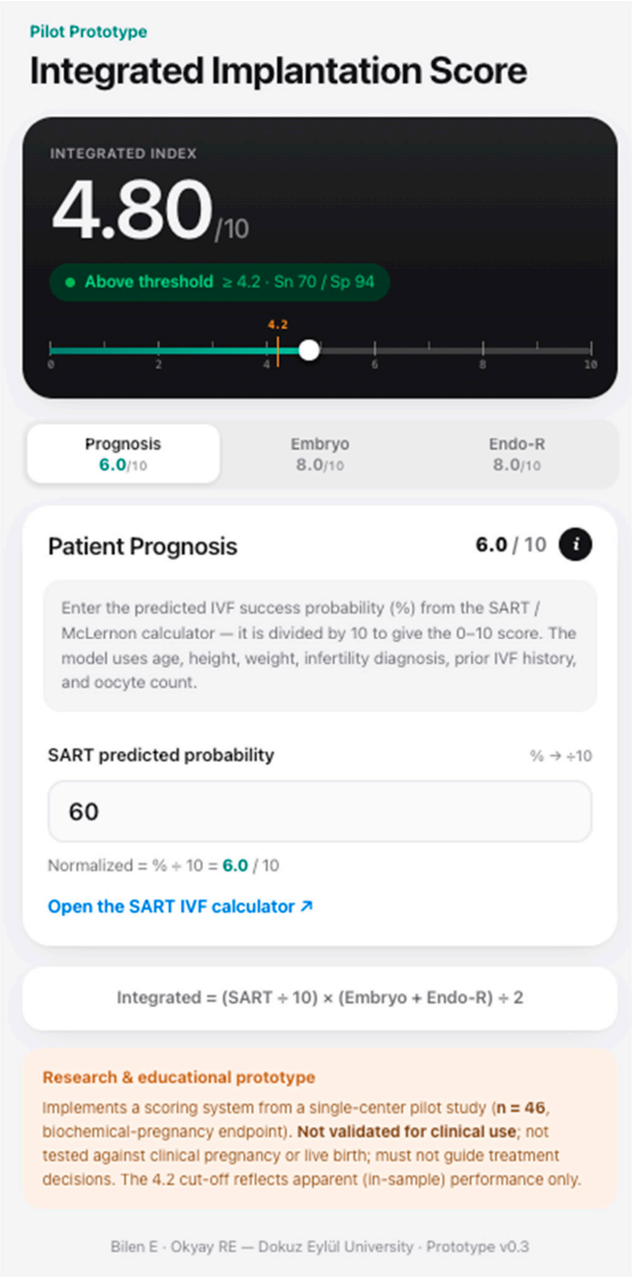

Screenshot of the browser-based prototype calculator implementing the Integrated Implantation Score. The tool is a research and educational prototype and is not validated for clinical use. Abbreviations: Endo-R, endometrial receptivity; SART, Society for Assisted Reproductive Technology.

Supplementary Methods S1

Rationale for the point allocations. Both embryo rubrics were built so that each stage-specific grading system spans the same 0–10 range, allowing a single Embryo Score to be used irrespective of transfer day. Within each system, the parameters were weighted in proportion to the emphasis they receive in the source grading scheme rather than by any data-driven optimization in this cohort.

For cleavage-stage embryos (Table 1), the five Baczkowski parameters were allocated a maximum of 2, 3, 3, 1 and 1 points respectively (cell number, fragmentation, symmetry, cytoplasmic appearance, zona pellucida), summing to 10. Fragmentation and symmetry carry the largest weights because they are the parameters most consistently associated with implantation in cleavage-stage grading; cytoplasmic appearance and zona pellucida carry the smallest because they are the least reproducible between observers. Where a parameter was recorded as a range, for example fragmentation grade 1–2, the midpoint of the corresponding point values was used.

For blastocysts (Table 2), the three Gardner parameters were allocated a maximum of 3, 3.5 and 3.5 points (expansion, inner cell mass, trophectoderm), again summing to 10. Inner cell mass and trophectoderm carry slightly more weight than expansion because they describe cell quality rather than developmental timing. Where expansion was recorded as a range, for example grade 5–6, the mean of the corresponding point values was used.

Four cycles had incomplete stage-appropriate morphological documentation and were assigned the cohort median Embryo Score; a complete-case analysis excluding these four cycles is reported in Table S3.

Calculator implementation. The calculator is a single self-contained HTML file with no server component, no external dependencies and no data transmission: all computation occurs in the browser and nothing is stored or sent. Each of the three domains is entered on its own tab. The SART Score is entered directly as a value between 0 and 10. The Embryo Score tab presents the parameters of Table 1 or Table 2 according to the transfer day selected, as radio buttons labeled with their point values, and sums them. The Endo-R tab takes endometrial thickness as a numeric input and the remaining four parameters as radio buttons, following Table 4. The three domain scores are combined in real time

using the pre-specified formula and displayed against the Youden-derived threshold of 4.2, with an on-screen statement that the threshold is an in-sample optimum from a 46-cycle pilot cohort and is not validated for clinical use.

The complete scoring rubric and source code are openly available at https://github.com/egehanbilen/endo-r-score, released under an open license so that a participating center can adopt the identical scoring without reimplementation.
